# An Update on Novel Therapeutic Warfronts of Extracellular Vesicles (EVs) in Cancer Treatment: Where We Are Standing Right Now and Where to Go in the Future

**DOI:** 10.1155/2019/9702562

**Published:** 2019-07-25

**Authors:** Muhammad Babar Khawar, Muddasir Hassan Abbasi, Zerwa Siddique, Amin Arif, Nadeem Sheikh

**Affiliations:** ^1^State Key Laboratory of Stem Cell and Reproductive Biology, Institute of Zoology, Chinese Academy of Sciences, Beijing 100101, China; ^2^University of Chinese Academy of Sciences, Beijing 100049, China; ^3^Cell & Molecular Biology Lab, Department of Zoology, University of the Punjab, Lahore, Pakistan; ^4^Department of Zoology, University of Okara, Okara, Pakistan; ^5^Centre for Applied Molecular Biology (CAMB), University of the Punjab, Lahore, Pakistan

## Abstract

Extracellular vesicles (EVs) are a heterogeneous group of membrane-bounded vesicles that are believed to be produced and secreted by presumably all cell types under physiological and pathological conditions, including tumors. EVs are very important vehicles in intercellular communications for both shorter and longer distances and are able to deliver a wide range of cargos including proteins, lipids, and various species of nucleic acids effectively. EVs have been emerging as a novel biotherapeutic platform to efficiently deliver therapeutic cargos to treat a broad range of diseases including cancer. This vast potential of drug delivery lies in their abilities to carry a variety of cargos and their ease in crossing the biological membranes. Similarly, their presence in a variety of body fluids makes them a potential biomarker for early diagnosis, prognostication, and surveillance of cancer. Here, we discuss the relatively least and understudied aspects of EV biology and tried to highlight the obstacles and limitations in their clinical applications and also described most of the new warfronts to beat cancer at multiple stages. However, much more challenges still remain to evaluate EV-based therapeutics, and we are very much hopeful that the current work prompts further discovery.

## 1. Introduction

A bidirectional communication exists between cells and their immediate surroundings that ensures the survival of cells and is an essential factor for both normal and pathophysiological circumstances. Traditionally, such crosstalk was believed to occur via the release of soluble cellular factors (i.e., chemokines, cytokines, and growth factors) [1–3] or via direct cell-cell contact; however, involvement of the extracellular vesicle (EV) in cellular communication has changed the notion over the past decade [4, 5]. Various eukaryotic cell types secrete these EVs *in vitro*, and their presence has been reported in a variety of body fluids including blood, bile, milk, and urine as well as in fecal matter [6]. A number of factors can induce the release of EVs, i.e., change in pH, stress, damage, irradiation, lack of oxygen, exposure to complement proteins, and also as a result of cell activation (i.e., platelet activation) [7, 8]. Secretion of EVs by plants and numerous pathogens including bacteria, Archaea, mycobacteria, and fungi is suggestive of an efficient evolutionarily conserved intercellular communication mechanism [9, 10].

Intercellular communication is an important phenomenon in multicellular organisms and usually mediated via direct contacts between the cells or via transfer of secretory molecules. Some of these secretory molecules are packaged into small lipid bilayer vesicles, known as extracellular vesicles (EVs), identified as a new means of intercellular communication. These EVs are major players in tumor progression and have shown a greater potential in therapeutic applications [11]. These diverse collections of vesicles are secreted by almost every type of cells [12]. Wolf identified these EVs for the first time in 1967 [13]. They successfully isolated minute particulate material from plasma free of platelets via ultracentrifugation. They originally named it as “platelet dust” [13] which was later on replaced by the currently known term “extracellular vesicles.” Afterward in 1981, cultured normal and neoplastic cells were found to secrete membranous vesicles which were suspected to take part in physiological processes [14]. In the following few years, a similar kind of small vesicles, named “exosomes,” was also found to be produced and secreted by reticulocytes *in vitro* [15]. Though only a little work was done in the early years following their discovery, recent rediscovery of EVs by cancer scientists has thrown the research into gear, and now, EV study is an exciting and rapidly growing field. The present term “EVs” was assigned by the International Society of Extracellular Vesicles (ISEV).

EVs play significant roles in multiple physiological processes including stem cell differentiation [16], autophagy [17], blood clotting [18], angiogenesis [19], immunity (innate and acquired) and immunomodulation [20, 21], pregnancy [22], embryo implantation [23], reproduction, placental physiology, semen regulatory function [24], and tissue regeneration [19]. Furthermore, the role of EVs in neuronal regeneration and in the development and functioning of the nervous system has also been anticipated as novel arbitrators of communication between the cells [25, 26]. Besides their contribution in normal physiology, EVs are also the key components in various pathologies like cancer [27–29] and development of multiple neurodegenerative diseases [30]. EVs mediate a variety of processes involved in cancer progression including inflammation, lymphogenesis, cell proliferation, epithelial-to-mesenchymal transition, angiogenesis, migration, suppression of the immune system, and metastasis—all of which are the so called “hallmarks of cancer” [31]. These extraordinary organelles have been associated with a number of aspects of cancer development and progression [32, 33], hence have a great potential to be used as biomarkers and ideal targets for novel future therapies for cancer treatment.

## 2. EV Diversity and Classification

**Figure 1:**
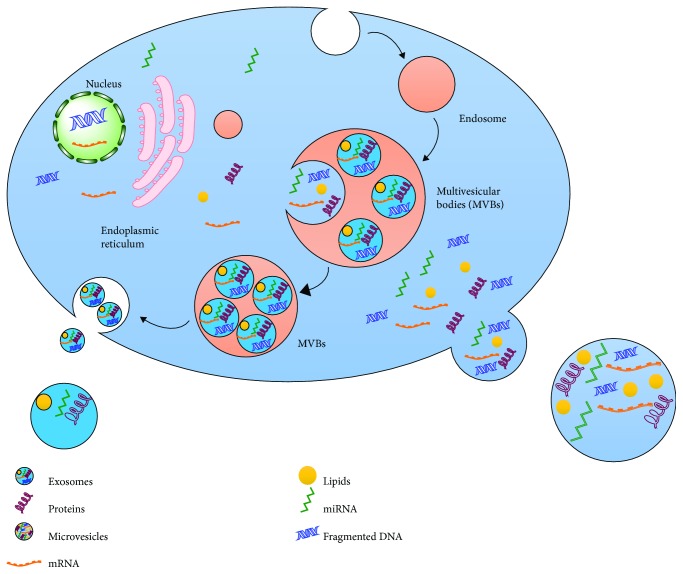
Biogenesis of exosomes and microvesicles: a schematic representation of endosome formation by internalizing the extracellular substances by invagination and pinching of the plasma membrane via endocytosis. These endosomes are transformed to multivesicular bodies (MVBs) by taking up a variety of cytosolic contents (proteins, nucleic acids, and various metabolites) via inward budding of late endosomes. Later, these MVBs may fuse with the plasma membrane at certain points to release the internal vesicles named as “exosomes.” In contrast, microvesicles are formed due to outward protrusion/blebbing of the plasma membrane. A diverse array of cargos is packed into these protrusions which pinched off the parent cell giving rise to microvesicles.

**Figure 2:**
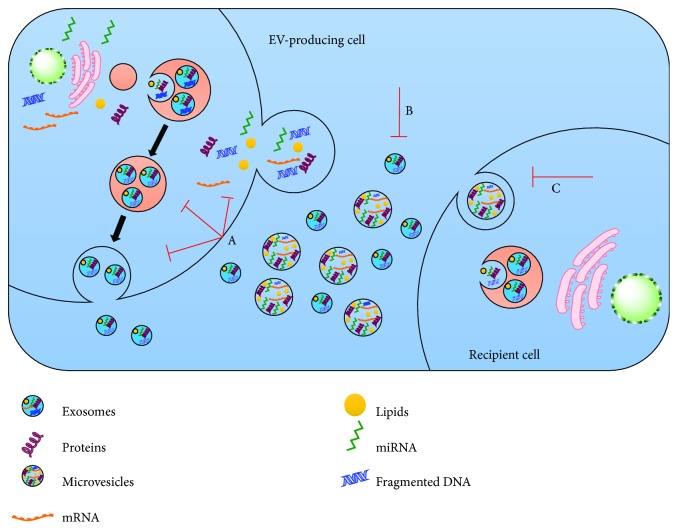
Therapeutic strategies to target EVs for cancer treatment. There are a number of potential ways to target the EV-mediated intercellular communication. (A) EV biogenesis or release can be targeted via interfering the specific components involved in EV production or surface shedding. (B) EVs can be targeted and specifically removed from the circulation using different substances, i.e., specific antibodies. (C) EV uptake/internalization by the recipient cells can be interrupted by targeting the EV ligands or cell surface receptors.

EVs are currently classified as exosomes, microvesicles, microparticles, ectosomes, oncosomes, and apoptotic bodies [34] on the basis of their size, origin, and characteristics [35] (Figures 1 and 2). However, this classification is considered insufficient to cover the heterogeneity that lies in cargo and their uncountable roles [36]. Exosomes are the best characterized and are less variable in size (ranging 40-150 nm) than other subtypes. Exosomes are produced as a result of membrane invaginations of endosomes resulting in the formation of multivesicular bodies (MVBs) and are stored as intraluminal vesicles (ILVs) that are secreted out of the cell once MVB fused with the plasma membrane at a certain point [37–39] (Figure 1).

Microvesicles (MVs), sometimes also referred to as ectosomes or microparticles or membrane particles, are larger and more heterogeneous in size (ranging from 100 nm to several microns) than exosomes [40, 41]. These cell surface-derived EVs are produced as a result of bulging of the plasma membrane and are ultimately shed from the cell surface as these blebs undergo fission upon proper stimulation [40, 41]. MVs are known to be full of phosphatidylserine and have several other lipid components [42]. MVs were firstly identified to be released from RBCs [43] and activated blood platelets [44] and were thought to contribute in the regulation of coagulation cascade [45]. Their formation is endorsed by increased Ca^2+^ levels that do so by altering the phospholipid distribution within the plasma membrane. A steady state or classical asymmetric lipid composition exists along the plasma membrane and is characterized by the existence of aminophospholipids towards the cytoplasmic side while phospholipids are on the side facing extracellular environment [46]. A variety of membrane enzymes including floppase, flippase, translocase, and scramblase help in the maintenance of this intricate balance. Increased intracellular Ca^2+^ in association with cytoskeleton modification [44] and recruitment of scramblase (a Ca^2+^-dependent enzyme) [47] relocate phosphatidylserine from the inner side to the outer side [48] in an ATP-dependent manner [49]. ADP-ribosylation factor 6- (ARF6-) mediated invasion and cytoskeletal remodeling in prostate and breast cancers are accused of the shedding MVs and all other classes of EVs [50] (Figure 1). Considering the heterogeneity in the size of MVs, the presence of numerous subpopulations can be speculated within this subdivision of EVs. The term “oncosomes” was coined to describe the MVs or EVs secreted by cancer cells [51]. It is noteworthy that the term “large oncosomes (LO)” is referred to as a specific class of EVs and used to describe a comparatively larger size (1-10 *μ*m) of subtype MVs, which originate directly from the plasma membrane, produced by cancer cells [52, 53].

Apoptotic bodies, range in size from 800 to 5000 nm, are produced during cellular blebbing and released by cells undergoing programmed cell death [41]. However, how they affect other cells is not well-studied yet.

EVs are usually classified on the basis of their origin; however, this classification and the current techniques employed for the identification of these EVs are insufficient to distinguish clearly each type of EV separately [54]. Some vesicles also originated from the nanotubular projections present on plasma membranes [55]. Recently, the presence of very small nanovesicles (8-12 nm) has been reported in peripheral blood [56] that was errantly separated along with exosomes. It would not be wrong to say that the current nomenclature has not been without debate, and size attributes of EV subtypes are ambiguous to fully distinguish them separately [6, 57]. As a matter of fact, the isolation techniques and protocols are still in the development process, and consensus best practices have not yet been achieved resulting in differences in EV subpopulations being evaluated between investigations [36, 58]. Therefore, the mode of biogenesis and cargo within EVs may prove crucial in establishing a more proper criterion for their classification. In line with this speculation, EV subpopulations have been characterized recently on the basis of their size, density, cargo, and their effects on target cells [36, 59, 60]. Therefore, new EV classes and modifications in the current classification criteria are expected to emerge as the field progresses. Additional vesicle types used to study lipid and protein behavior have also been reported, however not classified along with the above given vesicles. For instance, a number of stimuli, i.e., some chemicals (paraformaldehyde and dithiothreitol), salts, and laser treatment may result in the formation of giant plasma membrane vesicles (GPMVs) in live cells [61–63]. These vesicles are large sized and help in understanding the lipid and protein organization existing along the plasma membrane [62, 64, 65]. There are many other additional vesicle types including small, large, and giant uni- and multilamellar lipid vesicles [66] along with artificial plasma membrane vesicles [67] that are exclusively synthesized *in vitro*. These extraordinary vesicle species are employed therapeutically to enhance the stability and uptake of drugs and other biological molecules probably via the same mechanism adapted by natural EVs [68, 69]. Currently, most, if not all, cell types are believed to produce EVs [70, 71]; however, diseased states and stress not only upregulate their production but also alter the cargo contained within [72].

Considering the unexpected pace in the growth of EV-related information, Minimal Information for Studies of Extracellular Vesicles (“MISEV”) guidelines were proposed by ISEV five years ago and have been updated last year on the basis of evolution of the collective knowledge of the field in these past four years. These guidelines equipped the EV biologist with a collection of standardized protocols and procedures for a better documenting of EV-associated functions [34].

## 3. Therapeutic Approaches to Target EVs

As described before, EVs are major players in tumor progression via the transfer of cargo within them. As a matter of fact, the pathways targeted by EVs differ largely from conventional methods, i.e., chemotherapy or molecular targeting drugs. Therefore, three potential therapeutic approaches are proposed in this regard: (i) inhibition of EV formation, (ii) eradication of circulating EVs, and (iii) ablation of EV absorption [73] (Figure 2). A large number of investigations both *in vivo* and *in vitro* have stressed the efficacy of inhibiting EV production in cancer reduction. For instance, blockade of EV secretion and miR-210-3p transfer and subsequently suppression of angiogenesis and metastasis were observed, in a xenograft mouse model, as a result of nSMase2-knockdown [74]. In another study, repression of ovarian cancer dissemination, as a result of inhibition of EV production, was observed upon nSMase2-knockdown [75]. In addition to it, to date, a large number of other molecules involved in EV production, i.e., RAB27A, RAB27B, and TSG101, have been exploited to reduce the production of cancer-derived EVs [76, 77]. Although suppression of EV production seems quite an efficient strategy in cancer treatment, targeting genes involved in this pathway will affect many of the important biological activities of normal cells as they are the mode of intercellular communication [73]. For instance, nSMase2 has been found to express in normal neural cells [78]. Furthermore, downregulation of these genes showed different extent of their inhibitory effects on EV production among different cancer types. For example, there was no effect on EV production upon ablation of nSMase2 in the prostate cancer cell line [79]. Therefore, it is the need of the current time and would be a future challenge to identify genes associated with cancer type-specific EV production.

A novel therapeutic strategy to remove the circulating EVs was devised by Marleau and coworkers in 2012. A hemofiltration system was developed that was able to specifically trap the circulating cancer cell-derived HER2-expressing EVs [80]. These HER2-positive EVs hinder the available therapies and subsequently promote cancer development [81]; therefore, selectively targeting the HER-2-expressing EVs may prove a better approach in breast cancer treatment. In line with it, the circulating EVs have also been found to establish a premetastatic niche and subsequently promote cancer metastasis [33]. Hence, it can be speculated easily that elimination of these circulating EVs may help in the prevention of cancer metastasis. Recently, a new idea has been adapted to target circulating EVs in a human breast cancer xenograft mouse model [82]. In this study, a pronounced decrease in metastatic activity was observed upon the administration of anti-CD9 and CD63 (two of the most enriched receptors on the EV surface) antibodies [82]. However, there were found no prominent effects on primary site growth. Moreover, macrophages were utilized to internalize the EVs tagged by anti-CD9 and CD63 and were not allowed to promote cancer progression. Furthermore, more in-depth studies are required as anti-CD9 and CD63 antibodies are unable to selectively target the cancer-derived EVs in humans. However, identification of cancer-specific molecules on the EV surface and development of specific antibodies against them can likely help in eliminating the circulating EVs and subsequently prove effective in cancer treatment. Therefore, that investigation was believed to devise a novel treatment strategy for cancer.

Microvesicle cargo is simply internalized directly into the cytoplasm via plasma membrane-EV fusion while intact vesicles are taken up via several ways for transferring to the lysosomal or endosomal pathway [83–85]. The multiple ways of EV internalization include micropinocytosis [86, 87], clathrin and caveolin-mediated endocytosis, phagocytosis, and lipid raft endocytosis [88, 89]. In addition to it, another key factor that favors the fusion of EV membranes with the recipient cells is the low pH conditions produced by the tumor microenvironment [88]. Therefore, disruption of EV internalization may help in the formulation of new and better therapeutic approaches to prevent tumor progression and in cancer treatment. Recently, heparan sulfate proteoglycans (HSPGs) were found to act as a receptor of GBM-derived EVs [89]. A dose-dependent inhibition of EV uptake and a clear suppression of EV-dependent cell migration were seen in GBM in the presence of an HS mimetic, heparin. A number of molecules capable of EV internalization have been described to date, and many more are expected to be reported in the near future [84, 90, 91]. Despite the identification of several molecules responsible for EV internalization, the mechanism involved in this internalization is not very clear. However, caveolin-dependent endocytosis has been reported as a primary route for internalization of multiple myeloma cell-derived exosomes while some of the exosomes were taken up via macropinocytosis and membrane fusion [92]. Glycans are involved in energy storage and also serve as structural components that have been recently described to play an important role in several molecular recognition events. Glycans not only modulate recognition at the cell level but also regulate the intracellular traffic and folding of individual proteins [93, 94]. Abnormal glycosylation usually interrupts these crucial recognition events and may lead to cancer or other disorders like lysosomal storage diseases [94]. Although we are not familiar with the function of glycoconjugates in EV biology at present, several novel strategies that utilize EV glycosylation have already been emerging. Indeed, an extraordinary interest has been emerging recently in studying the effects of glycosylation modulation of EVs and their cargo. Glycoengineering is a promising field that is highly exploited to optimize the stability and alter the pharmacokinetics of protein-based drugs [95, 96]. This concept was proved for EVs by engineering Lamp2b protein (exosomal membrane protein) [97]. Furthermore, N-glycosylation was found to protect the peptides from lysosomal proteolysis [97]. In fact, a twofold increase in the efficiency of exosome delivery was found in the nervous system upon glycosylation of the desired peptides. Therefore, this strategy can be speculated the best choice to improve the uptake efficiency of peptide-targeted vesicles. Neuroblastoma-derived exosomes were found highly enriched in glycosphingolipids, and these glycans showed a huge therapeutic potential against Alzheimer's disease as they were capable of scavenging the *β*-amyloid [98]. The formation of hybrid exosomes having unique lipid components with the liposomes [99] has shown an enormous therapeutic potential of the glycolipid cargoes. These few experiments are the EV glycoengineering efforts that advocate a promising platform and future directions. Briefly, glycosylation can be exploited in manipulating the cargo protein recruitment and offers novel therapeutic targeting approaches [100, 101]. One more aspect that can be utilized to modulate the physicochemical characteristics of EVs is the sialylation status as it is capable of altering the vesicle charge [102]. This approach is yet to be achieved, but the availability of massive information on glycoengineering can be applied to EVs [103].

Unluckily, currently, there are no data available about the effects of inhibition of EV internalization under *in vivo* conditions. One of the prime reasons behind this insufficiency of investigations is that the cancer cell-associated EV uptake pathway is not quite clear yet. To ensure the integrity of normal cell homeostasis, it is necessary to fully understand the cancer-specific EV uptake pathways by the cancer biologists for therapeutic development. The presence of different EV protein markers has been recently described in different fractions of EVs; furthermore, these fractions were found to have different molecular and biological characteristics [36]. Likewise, immature dendritic cells have been found to release two EV subpopulations (small and large EVs) that were further found to affect the T helper cell in a different manner [104]. Therefore, identifying the EV subpopulations, their effects on target cells, and specific internalization pathway would be the best approach for better future therapies. On the basis of the above given contexts, inhibition of EV transfer could be speculated to be employed as a novel therapeutic approach in suppressing the tumor progression. Despite a great number of challenges, outstanding advances and pace in understanding of EVs are promising a better future [11].

## 4. EVs as Potent Novel Drug Delivery Systems

EVs have been emerging as attractive novel entities for drug delivery because of their structural analogy with liposomes [105]. Liposomes have proven their efficiency as a novel drug carrier and have been widely employed for drug delivery as they are very similar in composition with the plasma membranes [106]. Since that time, multiple commercialized liposome-based products like Myocet (an approved nonpegylated liposomal doxorubicin highly practiced against metastatic breast cancer), DaunoXome (an approved liposome employed against advanced HIV-associated Kaposi's sarcoma to deliver daunorubicin (DNR)), and Depocyt (approved against lymphomatous meningitis) have been introduced for therapeutic purposes [107]. Liposomal research has laid down the foundation to explore their physicochemical properties and stability for their employment as novel drug delivery agents [108–110]. Exploiting EVs is a better choice and more advantageous than liposomes as they are naturally produced by the cells and can easily transfer the desired drugs. These properties make EVs the best choice to be utilized as drug delivery agents even across the blood-brain barrier (BBB) [111]. Some of the potential EVs that have been used recently as drug delivery vehicles in different types of cancer are summarized in Table 1.

## 5. EVs in Cancer Treatment

A huge number of studies have provided the evidences of the use of these EVs as a splendid tool to deliver small interfering RNAs and other synthetic molecules for therapeutic purposes [123]. EVs have been employed in a number of animal model studies developed for different diseases as potent therapeutic DDSs [124]. Moreover, they are splendid antitumor DDSs as EVs are capable of passively targeting tumors because of their enhanced permeation and retention [125]. It is of great interest that genetically engineered EVs as targeted DDSs offer a dynamic and handy platform for specific and target-oriented drug delivery with better therapeutic outcomes. Recently, an efficient DDS was developed for the successful transfer of siRNA to the CNS via modified dendritic cell- (DC-) derived EVs [111]. The DCs, isolated from mice, were transected with a plasmid expressing EV surface protein, lysosome-associated membrane glycoprotein 2b (Lamp2b), along with rabies viral glycoprotein (RVG) that helps in binding with acetylcholine receptor. An efficient brain-targeting gene knockdown was observed by GAPDH siRNA-loaded DC-derived EVs, signifying their prospect as effective targeted DDSs. Effective delivery of both genes and proteins represents the potential of these extraordinary EVs to be served as cell-derived liposome-like nanoplatforms to cure various diseases including cancer [5, 40, 41, 126, 127]. Moreover, a zip code-like 25-nt sequence has been found to enhance the packaging of miRNAs into EVs and has pushed the research one step forward by guaranteeing high-yielding EVs loaded with various RNAs [128]. Amazingly, siRNA have also been utilized as therapeutics against tumors via bacterial outer membrane vesicles (OMVs). This study has highlighted the significance of bacteria in the production of biological nanovesicles and their application in drug delivery [129]. Furthermore, they are also being employed to transfer chemotherapeutic agents in addition to biomolecule-based drugs to enhance their efficiency and to minimize the possible side effects associated with them. For instance, an effective inhibition and successful reduction of breast and colon cancers have been achieved by encapsulation of the EVs with doxorubicin and curcumin [130, 131]. The outcomes of these investigations suggest that EVs offer an effective way to suppress cancerous tumors by delivering a wide range of the chemotherapeutics drugs. Recently, doxorubicin (chemotherapeutic drug) have been successfully delivered, via an i.v. injection, to *α*v integrin-positive breast cancer cells via exosomes isolated from Lamp2b-iRGD peptide expressing engineered immature mouse DCs (imDCs), and a remarkable suppression in tumor growth was observed [130]. Moreover, these therapeutic exosomes were less toxic and very effectual in cancer inhibition. Furthermore, their effectiveness in delivering the therapeutic cargo has also been authenticated employing multiple tumor models [132] including hepatocarcinoma [133], lymphocytic leukemia [134], and pancreas [135] and prostate [136] cancers.

Some of the potent EVs that have been utilized recently in different types of cancer are summarized in Table 2.

## 6. EV-Associated Antitumor ncRNAs

A number of attributes, including their release from the parent cells, delivery via the circulatory system, targeted cell uptake, and selective cargo transport, make them a promising and sizzling object for the selective drug delivery carrier [73, 144]. Therefore, investigators are proposing innovative and dynamic approaches for the modification of EVs specifically exosomes to cope with the current clinical challenges and therapeutic needs [145–147]. One of such approaches involves the direct modification of the contents of isolated exosomes. For instance, siRNAs and shRNAs have been incorporated into fibroblast-like mesenchymal stem cell-derived exosomes via electroporation to target KRAS^G12D^ mutation of pancreatic cancer [143]. Therapeutic use of natural exosomes is highly advantageous for several reasons compared to synthetic liposomes. For instance, exosomes are prevented from being phagocytosed by monocytes and macrophages due to the presence of CD47 on the exosomal membrane. Furthermore, the accumulation and uptake of exosomes by cancerous tissues are facilitated by some yet unknown native proteins present on the exosomal surface. Consequently, these “chimeric” exosomes were found to effectively execute an enhanced survival and reduced metastasis [143]. Another approach is to stimulate the parental cells to release modified exosomes. For example, exosomes containing miR-143 were obtained from MSCs pretreated with medium containing synthetic miR-143 and were found to successfully deliver these miRNAs to osteosarcoma cells to hamper their metastatic activity *in vitro* [148].

## 7. Application of EVs as Cancer Vaccines

Considering their production by every cell and their immune-modulatory effects, they can be employed for diagnostic purposes. Similarly, exosomes have shown antigen-presenting and immune-stimulatory potential and are being utilized for triggering antitumor responses. Moreover, release of exosomes from tumor cells is suggestive of their involvement in tumor microenvironments [149]. Cancer- and immune cell-derived EVs are capable of inducing immunostimulation to recipient cells. This prospect can presume the use of EVs as cancer vaccines, either derived from APCs or derived by tumors themselves [150]. These cancer-derived EVs are believed as potential proimmune elements because of the presence of several stimulatory molecules, i.e., heat shock proteins [151, 152] and numerous tumor antigens on their surface [151, 153, 154]. There is an ample amount of data available in favor of EVs as potent immune-suppressive agents [150, 155, 156]. Therefore, an encounter between the immune system and tumor EVs takes place in an immune-stimulatory vs. an immune-suppressive context [150], and by considering their immune-stimulatory features, these tumor EVs have been employed clinically as cancer vaccines [157]. In addition to a previous report of Kunigelis and Graner [150], another trial is also available on “clinicaltrials.gov” (NCT01550523). This trial was performed on patients who had resected the tumors and failed the prior therapies; an antisense construct against IGF1R was used to induce apoptotic cell death in autologous tumor cells. The cells were positioned in a biodiffusion chamber, and soluble ingredients, for the induction of immune response, were allowed to be released. Next, the chamber was inserted in the rectus sheath and was detached after 24 hours. Some of the subjects were found to develop deep vein thrombosis and were subjected to enoxaparin treatment to get rid of this problem. Except this minor trouble, the therapy was believed to be safe as some subjects were found to show complete response and some were found to show partial response under two (2) to twenty-seven- (27-) week timespan [158]. In the second phase I trial, the glioma cell-derived exosomes were referred to as immune stimulators by the authors [159]. The tumor-challenged mice were safeguarded via implanting chamber-based vaccine in this case probably due to the formation and release of antigen-bearing immunostimulatory exosomes. In line with these investigations, DC-derived EVs/exosomes (also referred to as dexosomes/DEX) have also been subjected to phase II trials [150]. For this purpose, multiple types of antigens (peptides, proteins, and tumor lysate) are loaded to DCs isolated from patients. Subsequently, the exosomes produced by the cells in the culture supernatants are utilized as cell-free cancer vaccines. Recently, inoperable non-small-cell lung cancer patients were subjected to chemotherapy followed by DEX-based immunotherapy for maintenance. For this purpose, MAGE/NY ESO/MART1 peptides were introduced in DCs via the pulse and cultured with gamma interferon (IFNG). Subsequently, DEX were isolated, and 1-27 injections of DEX were given to the patients. A median overall survival of fifteen (15) months along with median progression-free survival (PFS) of 2.2 months was found in the treated patients. An increased number of NK cells and an enhanced activity associated with NKp30 (NK surface ligand) were found in subjects with >2.2 month PFS [160]. It is of great interest that NK cell activation was found in an earlier trial [161]; in addition, an improvement of T cell responses but no induction of tumor-specific T cells was found upon IFNG addition. Upon cessation of chemotherapy, about 50% of the patients with PFS did not reach primary endpoint at 4 months with this trial; however, large-scale DEX production could be adapted to treat very advanced cancer. The updates about the use of either tumor-derived or immune cell-derived EVs to promote antitumor responses and cancer suppression are continuing to grow. A huge number of investigations are available in favor of tumor EV-driven immune suppression [150, 156, 162–164]. Inflammation is a major contributor in immune-mediated progression and tumor suppression [165], and nucleotide receptors are common mediators of inflammatory reaction [166] and cancer [167]. One of such receptor families is the purinoreceptor family that participates in immune responses mediated by EVs in immunity, inflammation, and cancer settings [168].

Radiotherapy is a new choice in enhancing the immunotherapy effects; however, radiations led to the oxidation, degradation, and accumulation of the DNA in the cytosol. This accumulation of DNA encourages the release of interferon-b from tumor cells via activation of the cGAS/STING-mediated DNA-sensing pathway. STING is a key signaling component that responds to pathogen-derived DNA by inducing the production of a variety of cytokines and type-I IFNs upon activation by its ligand, cyclic GMP-AMP (cGAMP). Cyclic GMP-AMP synthase (cGAS) associates with the pathogenic DNA and led to the formation of cGAMP from GTP and ATP. The resultant cGAS-STING axis stimulates the production of inflammatory cytokines and type-I IFNs via activating the NF-*κ*B and IRF3, respectively [169]. Interestingly, tumor growth was augmented during radiotherapy in the mouse model lacking STING because of the attenuation of antitumor T-cell activation [170, 171]. In addition to it, tumoral growth was restricted in a murine melanoma model upon intratumoral administration of cGAMP [172]. Therefore, the cGAS/STING signaling pathway is an attractive therapeutic approach in inducing the efficient immune responses against tumors.

In another study, tumor cell-derived microparticles (T-MPs) were described to be used as cell-free tumor vaccine recently. T-MP-based vaccinations were found effective against several tumor types, and T-MP-loaded dendritic cells (DC) were also found very fruitful in a number of tumor models [173, 174]. In these models, T-MPs efficiently delivered the DNA fragments to DCs that subsequently induced the expression of type I IFN via activating the cGAS/STING signaling pathway. Furthermore, the subsequent increase in the IFN level enhanced the antitumor immunity by promoting the presentation of tumor antigens to T-cells and maturation of DC. Indeed, this study represents a novel tumor cell-free vaccine strategy of high therapeutic potential [173].

## 8. EVs as Cancer Biomarkers

A substantial interest has been growing, in the past few years, in investigating the potential of tumor-associated EVs for diagnostic purposes and their exploitation for disease monitoring. EVs derived from a number of tumor types are believed to contain specific cargo including nucleic acid and various proteins [175]. The presence of tumor-derived EVs in circulating bodily fluids including cerebrospinal fluid (CSF), urine, and blood makes them an easy and readily accessible battery of biomarkers. Therefore, these tumor-derived EVs are speculated to be specifically served for longitudinal disease monitoring and early relapse detection [176]. A few of EV-associated cargo (particularly nucleic acid and proteins) are also capable of predicting the therapeutic response of a specific treatment. Collectively, EVs have been proven by a growing body of evidence as a new representative class of rich and readily accessible cancer biomarkers. Their potential as a cancer biomarker was explored for the very first time by comparing the contents of EVs derived from glioblastoma and from the cells of origin [32]. In this report, authors found tumor-specific RNA and protein species, reflective of the parental cell, enriched in the released EVs [32]. Accordingly, a vast assortment of tumor-specific species including various nucleic acid species such as lncRNA [177], miRNA [178], and mRNA [32, 179] and multiple posttranslational protein modifications [179] have been well recognized. The diagnostic and predictive values of these EVs have been utilized in multiple studies with different cancer types and further strengthened recently by the massive profiling of sixty cancer cell lines [175]. EV proteome, from all the tested samples, was reported to reflect the cellular proteome and transcriptome. EV proteomic data helps in their exemplification by hierarchical clustering and categorization of the basis of the originating cell [175]. This correlation between tumor-associated EVs and contents of secreting cells is highly important for brain and CNS-associated tumors where conducting a tissue biopsy is a limitation. For instance, an upregulation of miR21 was observed in glioblastoma multiforme-associated EVs in CSF compared to healthy controls [180, 181]. Moreover, a positive correlation was noted between the level of EV-miR21 and tumor burden. Consequently, a huge number of tumor-derived EV-miRNA having prognostic and diagnostic values have been described in other types of cancer including pancreatic [182], colorectal [183], and non-small-cell lung [184] cancers. In line with it, several candidate mRNAs (C-MYC, BCL-6, and PTEN), characterized with diagnostic value to predict progression-free survival, have been found in plasma-derived EVs from patients of non-Hodgkin's lymphoma [185]. Therefore, these reports have opened a new potential of tumor-associated EVs for noninvasive longitudinal disease monitoring [186]. The cancer-related EVs may also be helpful in early disease detection. For instance, it was shown in an *in vivo* pancreatic cancer model that particular EVs expressing a marker protein were upregulated even at the time when the tumor cannot be detected by conventional imaging techniques [187]. In addition to it, AML-EVs can be detected in the blood of acute myeloid leukemia (AML) patients even prior to the release of leukemic blasts in the blood [176]. Moreover, tumor-associated EVs have also been employed in the prediction of response to a specific treatment. Interestingly, tumor-associated EVs are capable of transferring resistance from drug-resistant to drug-sensitive cells via specific miRNAs and various protein species carried by them. Several therapies for a number of cancer types including pazopanib (chemotherapy) in soft tissue sarcoma [188], tamoxifen (antiestrogen) in breast cancer [189], and cetuximab (anti-EGFR) therapy in colon cancer [190] have been found to show the same resistance transfer phenomenon. Surprisingly, in all of these investigations, disruption of sensitivity to a specific drug and development of resistance were observed upon exposure to EVs from the resistant cells. Moreover, authors also illustrated a possible mechanism for Trastuzumab (anti-HER2) therapy in breast cancer [81]. Astonishingly, EV-associated HER2 was found to reduce the therapeutic effects of this drug as it is able to bind and decrease the available concentration of Trastuzumab [81]. Collectively, the above cited literature is suggestive of the prognostic, diagnostic, and predictive values of tumor-associated EVs [191].

Some of the potent EVs that have been recently found as potent biomarkers in different types of cancer are summarized in Table 3.

## 9. Conclusion and Future Perspectives

EVs are the potent carriers of cargo molecules including functional RNA species, many therapeutic agents like miRNAs, mRNAs, proteins, and peptides, and synthetic drugs. These small vesicles loaded with therapeutic agents are highly advantageous in terms of their biocompatibility, low immunogenicity, and innate ability to interact with target cells. Many futuristic approaches can be implicated from *ex vivo* and *in vivo* studies. However, due to complexity of EVs, many questions must be addressed prior to opting these molecules in clinics.

Advances in isolation and characterization techniques will allow more insight understanding and hence will provide a platform to develop EV-based therapeutic and diagnostic tools. An inexpensive, reliable method of isolations must be recognized and implemented to ensure that the optimal yield of EVs is being obtained in a safe and repeatable manner. Ultimately, a preferred method of isolating intact EVs must be identified and scaled so that EV-based options can be developed into a clinically viable therapy.

Upcoming research would completely get benefit of exosomes' ubiquitous occurrence in eukaryotic cells as they appear to provide an excess for anticancer therapy. Exosomes have been in attention for their role in the TME, and TDEs, in particular, provide a hopeful way for cancer remedies as mechanisms for superior drug delivery, tumor suppression, and immune regulation due to their appropriate dimension, composition, and homing capabilities. The progress of cancer encompasses the difficult and intricate communication of cells and signaling molecules in the TME, and exosomes have been shown to advance tumor growth through the inhibition of antitumor immunity and the development of angiogenesis.

Existing investigations in EVs are inadequate to the *in vitro* system. More *in vivo* studies must be conducted, like transgenic models of the breast cancer system, which helps us to have a better understanding of breast cancer cell-derived EVs. By *in vivo* imaging, we can know the source of EVs, their kinetics, numbers, the recipient cell types, and the even relationship between EVs and soluble factors. Efforts in this area to understand the biodistribution and bioavailability *in vivo* include elaborating the type and nature of interactions between EVs and the extracellular matrix and more pronounced *in vivo* models to test the relevance of *in vitro* observations. Improvement is being made here also, with growing *in vivo* imaging techniques enabling visualization of EV production and distribution *in vivo*.

To develop EV-mediated therapeutic, efficient, and scalable bioengineering solutions are required; again, progress is being made, but there remain technical challenges. Given the pace of advances in the EV field over the past decade, it is likely that rapid progress will be made in addressing these challenges, and the promise of EV clinical translation will begin to become a reality. We hope that in the forthcoming years, research and trials can make available more efficient EV-based therapeutic options.

## Figures and Tables

**Table 1:** Various EVs used as a drug delivery vehicle in disease treatment.

Serial no.	EV type	Loaded drug	Disease type	Remarks	References
1	DEXAFP	*α*-Fetoprotein	Hepatocellular carcinoma (HCC)	Improved tumor microenvironment, elevated CD8+ T lymphocytes expressing IFN-*γ*	[112]

2	miR-34c-5p-loaded exosome	miR-34c-5p	Acute myeloid leukemia (AML)	Downregulated miR-34c-5p, regulates its own expression by targeting RAB27B through positive feedback	[113]

3	(i) Doxorubicin-loaded breast cancer-derived exosomes (ExoDOX)	(i) Doxorubicin	Breast cancer	ExoDOX are more stable and tolerable compared to free drug cardiotoxicity	[114]
	(ii) IRGD-exos	(ii) Doxorubicin		The loaded specific IRGD-exos efficiently targeted *α*v integrin-positive breast cancer cells *in vitro*, showed slower tumor growth	[115]
	(iii) miR-let-7a-loaded GE11-positive exosome-dependent EFGR	(iii) miR-let-7a		Loaded exosome binds to PDGF receptor transmembrane domain of EFGR, causes tumor growth inhibition	[111]
	(iv) Tea polyphenol epigallocatechin gallate- (EGCG-) loaded exosomes	(iv) EGCG		Decrease in CSF-1 and CCL2 tumor growth factor-associated macrophages (M2), increased activity of tumor-inhibiting macrophage phenotype (M1), upregulation of miR-16	[116]

4	Macrophage-derived exosomes loaded with paclitaxel (exoPTX)	Paclitaxel	Multiple Drug Resistance (MDR) cancer	Increased drug toxicity by bypassing drug efflux transporter-mediated resistance mechanisms, decreased metastasis growth	[117]

5	(i) Loaded exosomes from brain endothelial cell line bEND.3	(i) Rhodamine, doxorubicin, and paclitaxel	Glioblastoma	Doxorubicin-loaded exosome showed higher activity and much reduction in tumor size in the brain of zebrafish	[118]
	(ii) Mesenchymal cell derived-exosomes loaded with anti-miR-9	(ii) Temozolomide (TMZ)		Reversion of multidrug transporter expression, sensitize cells to TMZ, increase caspase activity and cell death	[119]

6	Catalase loaded monocyte-derived exosomes (ExoCAT)	Catalase	Parkinson's disease (PD)	The exosomes prevented mononuclear phagocytic entrapment of drug	[120]

7	DC-derived Lamp2b fusion exosomes	siRNA against BACE1	Alzheimer's disease	Loaded exosomes expressed RVG surface peptide and actively targeted neuronal cells and specific gene silencing activity in targeted neurons	[111]

8	Pancreatic cancer cell-derived exosomes	Curcumin	Pancreatic cancer	Induced apoptosis	[121]

9	Curcumin-loaded exosomes (CUR-EXO)	Curcumin	Microglial cell inflammation	Inflammation reduced after 2 h, enhanced apoptosis. Intranasal administration caused inflammation-induced autoimmune encephalomyelitis in mice	[122]

**Table 2:** Different types of potential EVs in cancer treatment.

Serial no.	EV type	Cancer type	Mode of action	Remarks	References
1	DC-derived exosomes	Tumor-associated fibroblast	Immunotherapy	CD8+ T-cells releasing EVs kill mesenchymal stem cells (MSCs) and attenuate tumor growth	[137]

2	(i) Rab27a-regulated exosomes	Breast cancer	(i) Exosome release	(i) RNAi-dependent knockdown of Rab27a reduced exosome secretion, decreased tumor growth	[59]
	(ii) PEG-SMRwt-Clu regulated exosomes		(ii) Exosome release	(ii) Regulates secretion of Nef-positive exosome-like vesicles	[138]
	(iii) NK and DC-modulated exosomes		(iii) Exosome internalization	(iii) Impairment of DC differentiation by IL6 overexpression and Stat3 phosphorylation	[139]

3	Dimethyl amiloride- (DMA-) regulated exosomes	Acute myeloid leukemia	Exosome release	DMA inhibit exchange of Na+/H+ and Na+/Ca2+, improve efficacy of cyclophosphamide	[140]

4	(i) GBM-derived EVs	Glioblastoma	(i) Binding inhibition	(i) Heparin inhibits oncogenic EFGRvIII mRNA transferation	[141]
	(ii) Stromal cell-derived exosomes		(ii) Exosome overexpression	(ii) miR-302-367 in glioma cells overexpress exosomes	[113]

5	Prostate cancer cell-derived exosomes	Castration-resistant prostate cancer cell	Exosome release	Manumycin-A (MA) inhibits Ras/Raf/ERK1 signaling and ERK-dependent oncogenic splicing factor hnRNP H1 and release exosome in cancer	[142]

6	Exosomes	Pancreatic cancer	Delivery vehicle	The exosomes deliver RNAi to oncogenic KRAS	[143]

7	Colorectal cancer cell line-derived exosomes	Colorectal cancer	Exosome knockout	Amiloride inhibits exosome production and blunts MDSC suppressor functions	[140]

**Table 3:** Several potential EV biomarkers found in different body fluids.

Cancer type	Body fluid	Biomarker	Method/technique	Clinical significance	Reference
Breast cancer	Plasma serum	Integrins	ELISA	Src activation and upregulation of proinflammatory S100 genes	[100]
		miR-9	qRT-PCR		
		HER2, CD47, Del-1, miR-1246, miR-21	Microfluidic chip Adaptive dynamic artificial polyligand targeting (ADAPT)	Upregulated, miR-1246 attacks CCNG2 and promotes drug resistance against cancer	[192]
		Glutathione S-transferase P1 (GSTP1), ubiquitin carboxyl terminal hydrolase-1 (UCH-L1), NANOG, NEUROD1, HER2, KDR, CD49d, CXCR4, CD44, miR-340-5p, miR-130a-3p, miR-93-5p, miR-17-5p	qRT-PCR FCM PCR array	Partial remission (PR)/complete remission (CR) Progression-free survival (PFS) Disease-free survival (DFS) Distant organ metastasis Recurrence	[193]
		TRPC5	Confocal analysis, Western blot	Elevated levels show poor prognosis	[194]
		Survivin	ELISA	Elevated survivin-*Δ*Ex3 splice variant, while differential expression of survivin-2B in BC patients	[195]
		Periostin	Nanoparticle tracking analysis	Higher levels in patients with lymph node metastasis	[196]
		miR-373	qRT-PCR	Controversial effect on BC	[197]

Glioblastoma	Cerebrospinal fluid (CSF) Serum	EGFRvIII	Western blot	Mutated EGFRvIII cause: increase mitogenic factor Akt, suppress apoptosis, downregulate Bcl-2	[198]
		PTRF/caveolin-1, miR-21	qRT-PCR	Elevation leads to recurrence	[181]
		DNM3, p65, p53	Microarray	Elevated levels in primary and recurrent GBC	[199]

Melanoma	Serum Plasma	MDA-9, GRP78	Western blot	Elevation in metastasis	[200]
		miR-125b	Western blot	Melanoma patient PDS and OS patients showed high survival	[201]
		PD-1, CD28	qRT-PCR	Downregulation leads to MM progression	[202]
		MIA, S-100	Immunoaffinity capture	Interaction with ECM proteins: promote metastasis	[203]
		CD63, caveolin-1	In-house sandwich ELISA (Exotest)	Elevated levels in MP	[204]

Hepatocellular carcinoma (HCC)	Serum	TAK1	Microarray analysis	Upregulation	[205]
		miR-320a	qRT-PCR	Cancer suppression	[206]
		miR-122	qRT-PCR	Upregulate septin-9: taxol resistance	[207]
		miR-21, 211, 222, 224	qRT-PCR	Upregulation in cancer patients	[208]
		miR-718, 1246	qRT-PCR	Downregulation causes HCC progression, miR-718 targets EGR-3 and increases proliferation	[209]

Ovarian cancer	Plasma Serum	miR-21, 141, 200a, 200c, 200b, 203, 205, 214, 222-3p	miRNA array	Suppress apoptosis through binding to APAF1, grant paclitaxel resistance. miR-21 targets Bcl-2, TPM1, PDCD4, maspin, and PTEN leading to tumor proliferation. miR-200 attacks ZEB1/2 leading to EM. miR-205 targets the HER2 pathway causing tumor suppression	[210]
		Phosphatidylserine	Nanoparticle tracking analysis	Elevated levels in cancer patients	[211]
		Claudin-4	Western blot	Upregulation in OC patients	[212]

Multiple myeloma	Serum	MSC-derived miR-15a, Let-7b, miR-18a	qRT-PCR array analysis	Suppressor of MM Elevated levels in cancer patients	[213]

Colorectal cancer (CRC)	Serum	miR-9	qRT-PCR	Inhibits suppressive expression SOCS5, upregulate endothelial cell migration	[214]
		CRNDE-h, miR-19a-3p, 21-5p, 425-5p, 17-92a	ExoScreen	Upregulation in cancer patients	[215]
		miR-4772-3p	qRT-PCR	Lower level causes cancer recurrence	[216]
		let-7a, miR150, 1246, 1229, 223, 21, 23a	qRT-PCR	let-7a binds KRAS and inhibits cancer, miR-21 downregulates p53	[217]

Prostate cancer	Serum Urine Plasma	LncRNA-p21, miR-21, 375 miR-1290	qRT-PCR	Elevation in prostate cancer patients	[218]
		PSA, PSMA, P-glycoprotein	qRT-PCR	Elevation causes castration-resident prostate cancer Elevation in docetaxel-resistant patients	[219]
		miR-1246	Western blot	Overexpression leads to positive metastasis	[220]
		TM256, LAMTOR1, VATL, ADIRF, survivin	qRT-PCR ELISA	VATL increases metastasis, LAMTOR1 regulates mTOR signaling	[221]

Pancreatic cancer	Serum	Glypican-1 miR-1246, 4644, 3976, 4306	GFP qRT-PCR	Increased levels in pancreatic cancer patient's upregulation in 83% of pancreatic cancer patients	[222]
		CD44, V6, Tspan8, EpCAM, CD104	ELISA	CD44v6 target MET and VEFGR-2 pathways to promote metastasis	[223]

Non-small-cell lung cancer (NSCLC)	Plasma	miR-302c, 302a, 126	Western blot	Elevation in cancer patients	[224]
	Bronchoalveolar lavage	EML4-ALK	Electron microscopy		
		miR 181-5p, 30a-3p, 361-5p, 15b-5p, 320b, 30e-3p	Nanoparticle tracking analysis		
		EpCAM, NY-ESO-1, Alix, PLAP, miR-24	miRNA-seq	EFGR mutation leads to metastasis, miR-24 targets Jab1/CSN5 to promote tumorigenesis	[225]

Acute myeloid leukemia (AML)	Plasma	CD34	Immunoaffinity capture	Elevated CD34+ exosomes in AML patients	[226]

Cervical cancer	Cervicovaginal lavage specimens	miR-21, 146a	qRT-PCR	Elevation in HPV+ patients	[227]
		Survivin	qRT-PCR	Suppress genotoxic-induced stress apoptosis and enhance proliferation	[228]

Bladder cancer	Serum Urine	Lnc-UCA1	qRT-PCR	Elevated expression in BC patients	[229]
		EDIL-3	Western blot	Tumor progression via activation of EFGR	[230]
